# Examining the Dynamics of COVID-19 Misinformation: Social Media Trends, Vaccine Discourse, and Public Sentiment

**DOI:** 10.7759/cureus.48239

**Published:** 2023-11-03

**Authors:** G. V. R. Meghana, Durga P Chavali

**Affiliations:** 1 Public Health Dentistry, Panineeya Mahavidyalaya Institute of Dental Sciences and Research Center, Hyderabad, IND; 2 Information Technology Applications, Trinity Health, Livonia, USA

**Keywords:** social media, misinformation, trend, sentiment analysis, anti vaccine, common hashtags, common search terms

## Abstract

Introduction: COVID-19, known as coronavirus disease, has prompted a global reevaluation of societal norms. The World Health Organization (WHO) declared it a worldwide Public Health Emergency on January 30, 2020. Subsequently, governments and pharmaceutical firms developed vaccines, such as mRNA options from Pfizer and Moderna, alongside viral vector alternatives to combat the escalating COVID-19 case tally. Extensive inquiry was directed toward assessing vaccine efficiency. Nonetheless, vaccine discourse has surged across social media, prominently involving the anti-vaxxer community. This faction’s hesitancy, rooted in reservations about efficacy, potential side effects, and conspiracy notions, contributes to an ongoing dialogue.

Objective: This investigation delves into social media’s role in proliferating COVID-19 misinformation, utilizing tools like Python, Excel, and external resources to craft data visuals that elucidate trends influencing misinformation dissemination and its hypothetical ties to elevated COVID-19 cases. Scrutiny of Twitter trends illuminates the prevalence of the hashtag #covidvaccine, although the platform curbs anti-vaccine hashtags.

Result: Analysis of sentiment across 207,006 tweets reveals a prevailing positive sentiment toward COVID-19 vaccines, coexisting with lingering skepticism. Google trends reflect increased anti-vaccine ideology queries, notably post-FDA vaccine approval in December 2020, indicating public doubt.

Conclusion: While limitations encompass data granularity, geographic origins of false tweets, bot account quantification on Twitter, and comprehensive digital resources, this study pioneers reference for forthcoming investigations. Its objective is to mitigate the diffusion of misinformation.

## Introduction

The news of the coronavirus outbreak began on December 31, 2019, and the World Health Organization (WHO) declared it a Public Health Emergency of International Concern on January 30, 2020 [1]. This disease, commonly known as COVID-19, induces severe acute respiratory syndrome (SARS) [1], leading to symptoms such as coughing, shortness of breath, or body temperatures exceeding 38 degrees Celsius [2]. The primary method for COVID-19 testing is through a nasal mid-turbinate (NMT) swab. If a test comes back positive, individuals are required to self-isolate for 14 days, with close contacts following the same protocol [2,3]. Due to the rapid global spread of COVID-19, it was declared a pandemic on March 11, 2020 [1]. This underscores the significant impact of this phenomenon, which has brought about profound physical and mental consequences for those affected. It has also reshaped societal norms, leading to remote work and schooling, as well as the adoption of mask-wearing practices. During this critical period, with the world in need of strong leadership, the response of governments and influential organizations has been a topic of controversy, prompting individuals to develop personal biases regarding the actions of health organizations [4].

The most significant response to combat COVID-19 has come through vaccination programs. When COVID-19 was declared a pandemic, global health organizations, pharmaceutical companies, and governments took action to develop vaccines capable of generating a robust immune response to the virus. Messenger RNA (mRNA) vaccines were among the first authorized for use in the United States, according to the Centers for Disease Control and Prevention (CDC) [5]. Unlike traditional vaccines, which introduce a modified virus, mRNA vaccines instruct cells to produce a “spike protein” to trigger an immune response. When the immune system detects this foreign protein, it produces antibodies, effectively training the body to defend against a future COVID-19 infection. This method offers the advantage of long-term immunity. Notable mRNA vaccines in North America include Pfizer and Moderna, both of which require two doses administered approximately three to four weeks apart [5]. While other COVID-19 vaccines, such as AstraZeneca and Johnson & Johnson, exist, they are not being administered in Canada due to growing health concerns. Vaccine effectiveness and safety have been sources of controversy, with concerns about side effects and the rapid development process. A significant portion of the population is hesitant to receive the COVID-19 vaccine, citing concerns about ingredient purity and potential side effects [6]. A study suggests that an individual's decision to receive the vaccine is influenced by the approval of the vaccine within their social circle, including peers and network connections [6]. Distrust in vaccine-producing corporations, often seen as prioritizing profit over safety, and skepticism toward policymakers have further contributed to vaccine hesitancy [6].

The advent of the Internet and social media has accelerated the spread of information. Our interconnected world has become a global community where information, creativity, and perspectives transcend borders. Social media's role in shaping public opinion has become increasingly powerful, giving individuals a platform to advocate for various causes, address societal imbalances, respond to COVID-19-related issues (such as anti-mask campaigns), and champion public rights, as exemplified by the Johns Hopkins movement. In this digital age, information, whether accurate or not, can reach millions within seconds.

Notably, the spread of misinformation concerning COVID-19 vaccines has been rampant [7]. Some common misconceptions are outlined below. One prevalent misconception is the belief that scientists rushed the development of the COVID-19 vaccine, raising concerns about its effectiveness and safety. Contrary to these misconceptions, extensive research has demonstrated an impressive 95% efficacy rate for the vaccine and has not reported any severe or potentially fatal adverse reactions, as confirmed by Johns Hopkins [8]. Another misconception suggests that receiving the COVID-19 vaccine eliminates the need for mask-wearing. However, the CDC has updated safety guidelines for fully vaccinated individuals, allowing them to resume mask-free activities and normal social interactions, except where legal requirements dictate otherwise, as reported by Johns Hopkins [8]. Another widespread misconception revolves around the notion that the vaccine's side effects pose a significant risk. In reality, the CDC affirms that while Pfizer and Moderna COVID-19 vaccines may produce side effects, the overwhelming majority are brief and non-severe, indicating the vaccine's active engagement with the immune system [8].

In contemporary society, social media serves as a powerful catalyst for spreading both accurate and inaccurate information. Individuals with substantial influence on social media often share unverified information on topics outside their expertise [9]. Social media provides a platform for open expression of opinions, but its lack of information filtering can fuel vaccine hesitancy by disseminating misinformation and polarizing public opinion. Since the onset of the pandemic, false information has circulated, including claims of vaccine-related deaths, the virus being a government conspiracy or a bioweapon. While many hold positive views of vaccines, a group known as “anti-vaxxers” presents a challenge for health officials tasked with debunking misinformation and conveying facts to the general public. Studies have shown that exposure to misinformation through social media reduces people's willingness to get vaccinated [10]. Vaccination campaigns face difficulties in persuading individuals who have already encountered misinformation [10]. Once preconceived notions take hold in the public consciousness, they become challenging to undo [10].

This paper investigates the impact of the proliferation of anti-vaccine sentiments on the rise in COVID-19 cases. We analyze data obtained from Twitter and Google Trends and apply coding software for data analysis. Our hypothesis suggests a significant prevalence of search terms and hashtags associated with anti-vaccine perspectives on digital platforms, potentially exerting substantial influence on public perceptions of vaccines.

## Materials and methods

This study aimed to comprehensively investigate and establish the correlation between the dissemination of vaccine-related misinformation, the notable decline in the administration of vaccine doses, and the concurrent surge in COVID-19 cases. In pursuit of this objective, a multifaceted approach encompassing diverse data collection and analysis methodologies was adopted.

A comprehensive data collection process was initiated, incorporating various sources, including the vaccine database procured from Kaggle, dynamic data from Google Trends, and relevant statistics obtained from reputable sources like Google and other reliable websites. The data were meticulously examined and analyzed to extract crucial insights into the prevailing trends associated with vaccines and the evolving landscape of COVID-19 cases. Leveraging the capabilities of Google Collab and the versatile Panda library, the collected datasets were systematically processed and analyzed to discern patterns and trends relating to the prevalence of specific anti-vaccine sentiments.

The application of Google Collab, an interactive cloud-based development platform, facilitated the seamless analysis of complex datasets from the Kaggle vaccine database. Leveraging the robust functionalities of the Panda library, which offers extensive data manipulation tools, enabled the effective extraction and interpretation of crucial information encoded within the dataset. Specific algorithms and data parsing techniques were employed to identify and quantify the occurrences of distinct anti-vaccine hashtags, providing valuable insights into prevalent sentiments and discussions pertaining to the vaccine landscape.

The integrity and accuracy of the collected data were paramount to the study’s credibility. As such, an intricate process of data processing and cleansing was undertaken, meticulously filtering out extraneous information and focusing on pertinent parameters that elucidated the prevalence of anti-vaccine sentiments. Rigorous data scrubbing techniques were applied to ensure that the dataset used for analysis was devoid of any inconsistencies or biases, thereby enhancing the robustness of the study’s findings.

We utilized a dataset from Kaggle's vaccine database to identify common vaccine-related hashtags. Subsequently, we processed the data using Google Colab. Specifically, the CSV files retrieved from Kaggle were analyzed using the Pandas library to extract and tally occurrences of specific anti-vaccine hashtags, which were then presented as can be seen in Table 1.

**Table 1:** Usage of common hashtags by individuals on Twitter.

Common Hashtags	Number of Occurrences in Tweets
#covidvaccine	113337
#antivaccine	33
#vaccineskill	2
#antivaxxers	134
#chip	46
#covidisfake	1
#autism	33
#mutation	39
#notsafe	4
#antivaxx	195
#vaccineinjury	27

**Figure 1:**
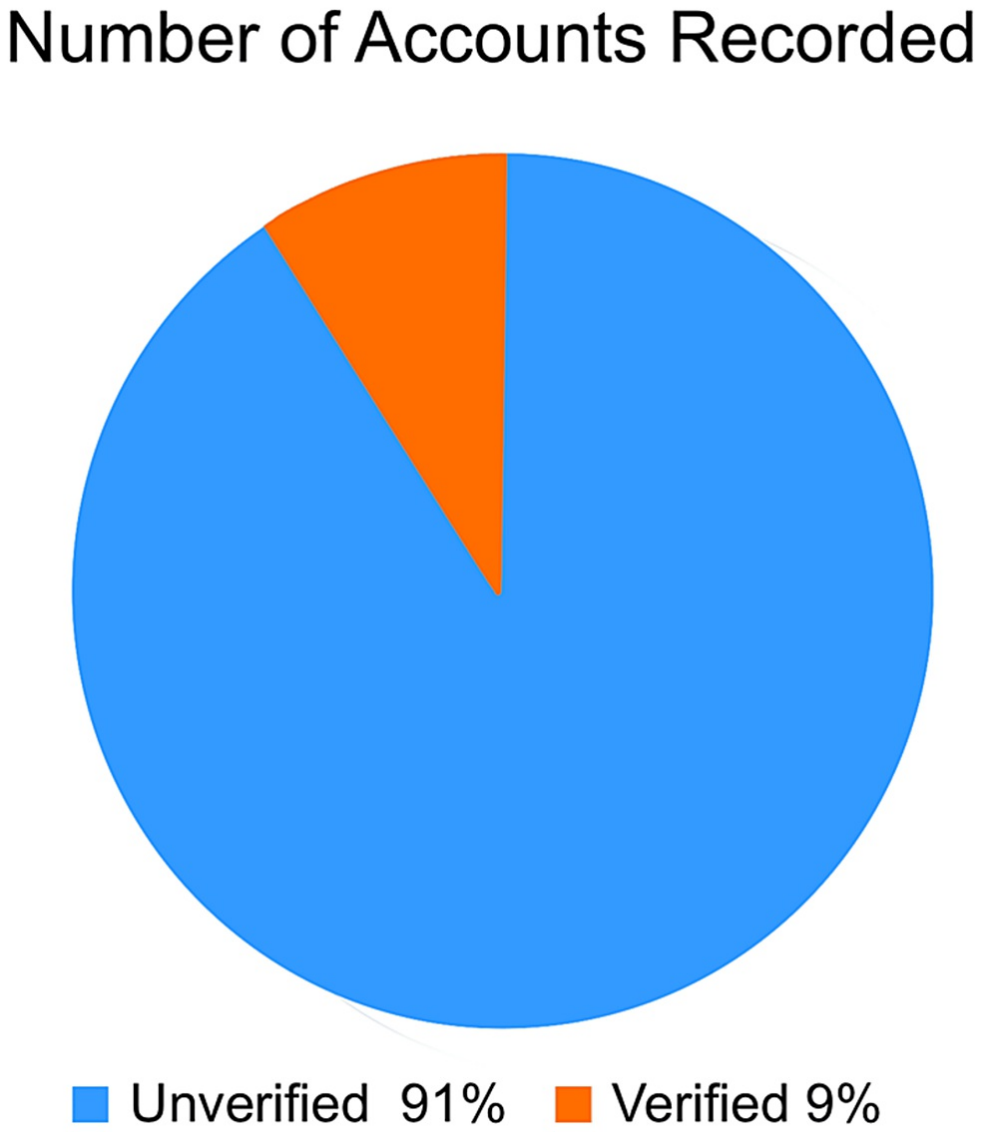
Number of accounts verified versus unverified on Twitter.

The construction of a comprehensive pie chart (Figure 1) was instrumental in visualizing the distribution of various accounts actively participating in vaccine-related discourse. Leveraging data extracted from the vaccine database, the pie chart effectively highlighted the proportions of verified and unverified accounts, providing crucial insights into the landscape of vaccine-related discussions and the potential influence of different account types on disseminating vaccine-related information.

Google Trends data was a dynamic and informative resource for discerning prevalent search trends and user interests concerning anti-vaccine sentiments. These data were analyzed alongside the patterns of vaccine dose administrations and the parallel escalation in the prevalence of COVID-19 cases. The correlation between trending search queries and public sentiments was meticulously examined, shedding light on the evolving public perception and attitudes toward vaccine-related information dissemination.

**Figure 2:**
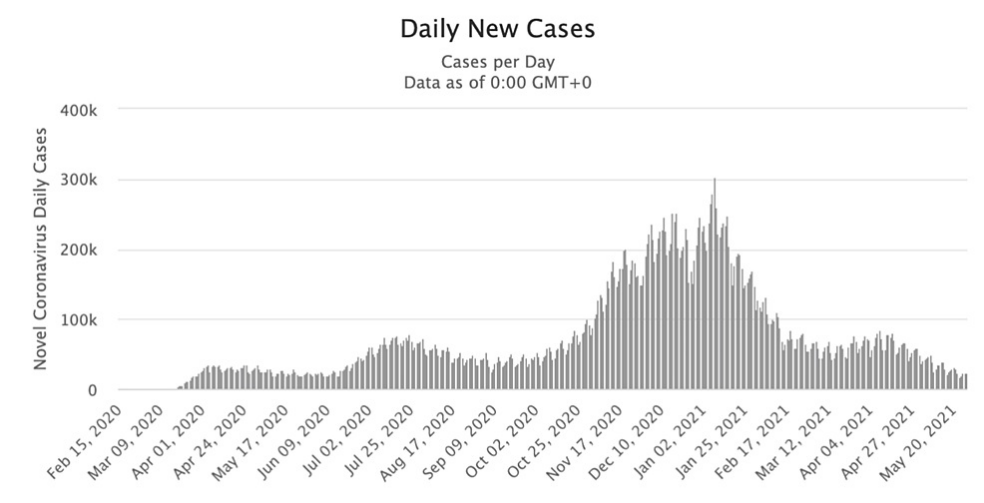
A timeline of the number of COVID-19 cases in the United States of America from February 2020 to May 2021.

**Figure 3:**
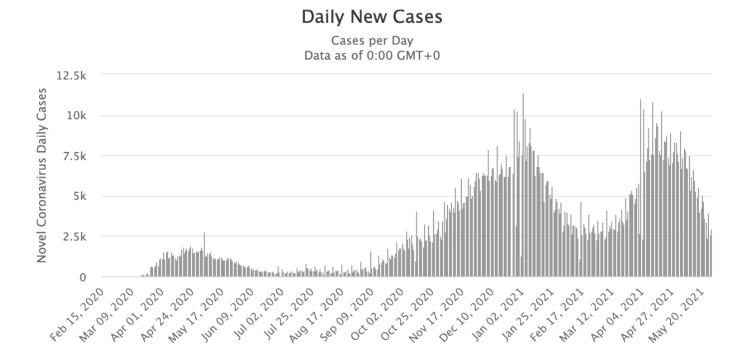
A timeline of the number of COVID-19 cases in Canada from February 2020 to May 2021.

**Figure 4:**
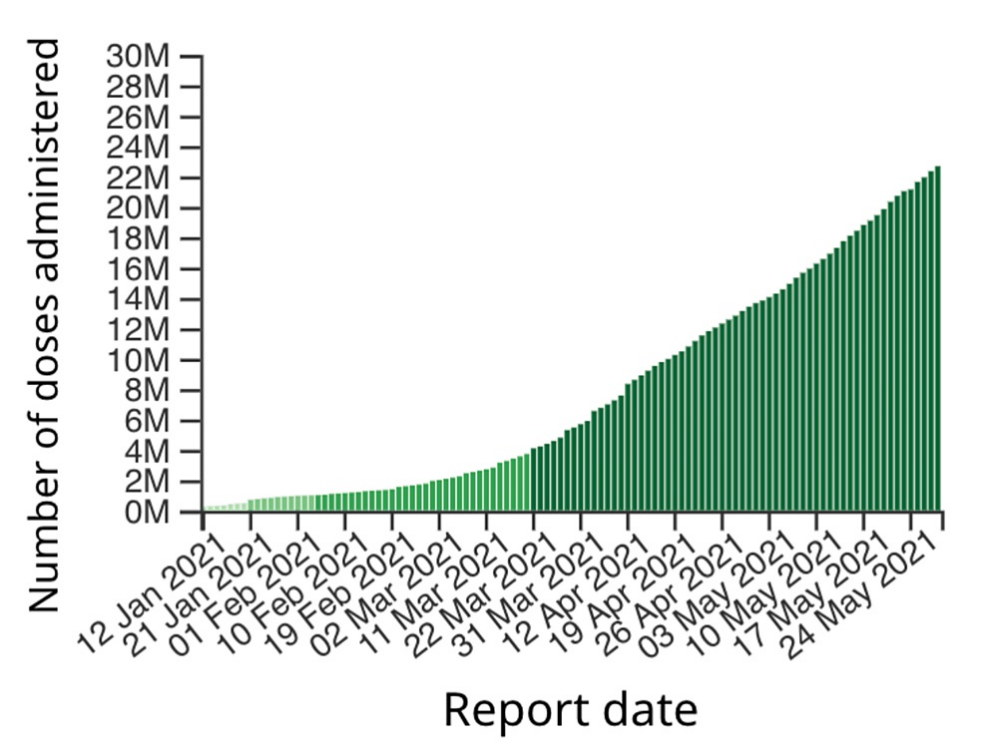
Cumulative number of COVID-19 vaccine doses administered from January 2021 to May 2021 in Canada.

**Figure 5:**
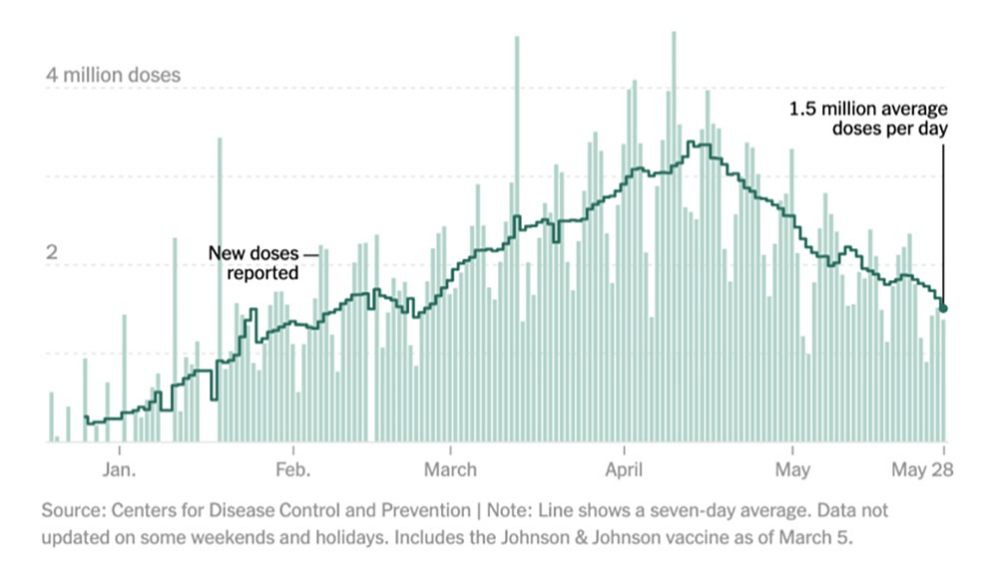
Number of COVID-19 vaccines administered in the United States of America from January 2021 to May 2021.

Figures illustrating the progression of COVID-19 cases over time in the United States (Figure 2) and Canada (Figure 3) were obtained from credible sources, including Google. Additionally, Figures 4, 5 depicted the vaccination trends in Canada and the US, respectively, allowing for correlation analysis between COVID-19 cases and vaccine administration. These graphs served as crucial visual aids in mapping the correlation between the prevalence of COVID-19 cases and the corresponding patterns of vaccine dose administrations, thereby offering a comprehensive perspective on the dynamic interplay between vaccination efforts and the trajectory of the pandemic. 

Employing advanced data analysis tools and methodologies such as Python and R, the collected datasets underwent a rigorous analysis process to extract nuanced insights and meaningful correlations. Python was utilized to clean and preprocess the data from Twitter and Google Trends. This included removing duplicate tweets, removing stop words, and stemming and lemmatizing the text. Regression analysis in R was utilized to discern patterns, trends, and relationships within the data, facilitating a comprehensive interpretation of the complex interplay between vaccine-related sentiments and the dynamics of COVID-19 prevalence. The rigorous data analysis process was essential in ensuring the credibility and reliability of the study’s findings, offering valuable insights into the multifaceted dynamics governing the relationship between vaccine misinformation and the trajectory of the pandemic.

## Results

Number of COVID-19 cases in the US and Canada

As seen in Figure 2, there is a spike in cases of COVID-19 in the United States between November 2020 and January 2021, peaking at 300,000 cases per day. This was also the time when the Pfizer-BioNTech COVID-19 Vaccine and Moderna COVID vaccine were discovered and eventually approved by the FDA. The surge in the number of cases shows a slow decline from February 2021. Canada shows a similar trend as the US between November 2020 and January 2021, as shown in Figure 3, peaking at around 11,000. While the daily cases drop slightly during February, there is a drastic increase in the rate of daily occurrences from March till date.

Number of people receiving the COVID-19 dose of vaccination 

Figure 4 shows a rising trend in the number of doses administered in Canada from January 2021 to May 2021. The rate of vaccine administration was a slow process in the beginning. From January to March, only about four million dosages were administered. However, this rate started drastically increasing toward the end of March, and by the end of May, a little above 22 million people were vaccinated. In the USA, a more significant number of doses were administered per day in comparison to Canada, and the rate of vaccination was faster as well. By mid-January, the US could vaccinate about one million people on average daily. Over time, a rapid increase in the trend can be noticed, which peaked at 4.5 million doses per day around mid-April. From that point, the rate of dosage administration shows a declining trend. 

Number of accounts verified versus unverified on Twitter 

To analyze the number of verified accounts on Twitter compared to that of the unverified account, a total of 206,993 accounts were analyzed. It was found that 187,533 accounts, which is 91% of the total data, were unverified, whereas 19,460 accounts, or 9%, were not verified, as shown in Figure 1. While the data are not directly related to COVID-19 vaccine misinformation, it is hypothesized that some unverified accounts are controlled by bots that can potentially be programmed to spread false information. Due to limitations in regard to accessible data, it was not possible to confirm the exact number of bot profiles.

Usage of common hashtags related to anti-vaccine ideology 

A total of 200 COVID-19-related hashtags were analyzed, out of which the most-used and relevant ones are reported in this paper. Before analysis, a moderate to large amount of occurrence for anti-vaccine hashtags was expected. While there are many searches for COVID-19 vaccine-related information, the limited number of occurrences of anti-vaccine and vaccine misinformation-linked hashtags was an unexpected discovery. Upon further investigation, it was found that social media platforms like Twitter, Instagram, etc., block the use of search terms that are associated with misinformation spread, limiting the rate of spread to an extent. 

Sentiment analysis in relation to COVID-19 vaccine on Twitter 

The COVID-19 vaccine discovery evoked a wide range of emotions among the general population. A sentiment analysis conducted on Twitter provides information about people’s sentiments toward vaccines. The sentiment score depends on the Python library used to analyze the tweets for sentiments by Durga P. Chavali from Kaggle. A total of 207,006 tweets were analyzed, and it was found that a significant number of users had a positive reaction and a reaction of joy, anticipation, trust, and surprise toward the COVID-19 vaccines. However, many tweets displayed adverse reactions, including feelings of anger, fear, disgust, and sadness. Many negative tweets are very likely to contribute to the spread of more false information and hesitancy toward the vaccine. 

Timeline of use of common search terms related to anti-vaccine ideology on Twitter 

**Figure 6:**
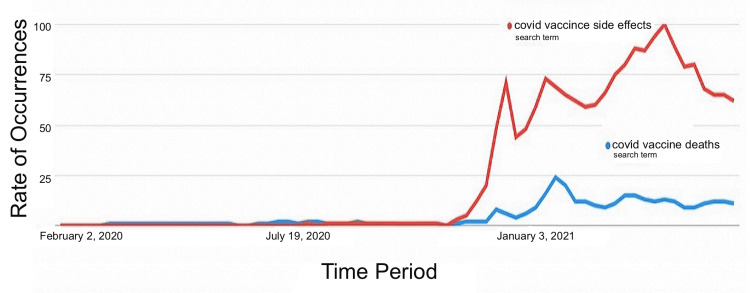
Rate of occurrences of search terms “covid-vaccine side effects” (red) and “covid-vaccine deaths” (blue) at different periods from February 2020 to May 2021.

**Figure 7:**
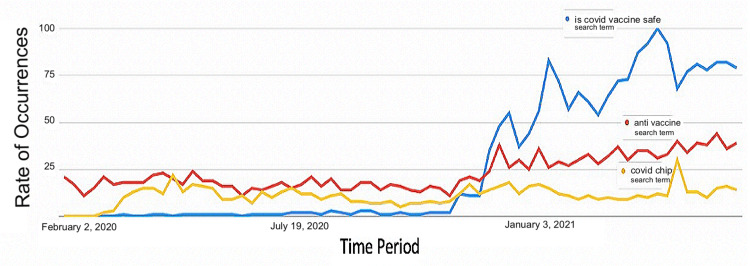
Rate of occurrences of search terms “is covid vaccine safe” (blue), “anti-vaccine” (red), and “covid chip” (yellow) at different periods from February 2020 to May 2021.

A total of 100 standard search terms used worldwide that are related to COVID-19 anti-vaccine ideology were analyzed, out of which the five most frequently used terms are reported in Figures 6, 7. The others were excluded from the list due to the extremely low rate of occurrence. A common observable trend between these five search terms is that the frequency of occurrence has increased since December 2020, which is also when the COVID-19 vaccines were approved. Figure 6 shows that people are most likely to search for the side effects of the COVID-19 vaccine instead of the rate of mortality caused by it. Figure 7 confirms that many people were concerned with the safety of the vaccines, which is inferred from the frequent use of the search term “is COVID vaccine safe?”. A large group of individuals were also against the administration of the COVID-19 vaccine and were actively seeking information about “anti-vaccine” ideology. The spread of misinformation is also evident from the rate of occurrence of the search term “COVID chip,” implying the fact that a significant number of individuals were concerned with a microchip being injected into their bodies through the vaccines.

## Discussion

The overall purpose of this study was to examine the anti-vaccine ideology that has spread its influence across various social media platforms, leading to public health misinformation. Statistical data has been collected across various sources, such as Google Trends, Twitter, etc., that examine the correlation between COVID-19 incidence rates post-development and FDA approval of the administration of the mRNA COVID-19 vaccine. Upon examining the obtained results, it can be concluded that a relatively large proportion of the population was resentful and fearful of being administered the COVID-19 vaccine. This fear and choice to not receive the vaccine stems from a multitude of factors. This includes a lack of trust in the efficacy and ingredients, the fear of adverse symptoms, and the idea of “politicized medicine" [5]. This fear arises from public health information that stems from social media.

Over the past century, the development of social media platforms has revolutionized the vast array of locations where individuals access online information. The controversy behind the accuracy of information on social media has been a hot topic due to the relevance of bias that is brought in from online information from these sources. Public health misinformation is defined as a health-related claim or fact that is false based on current scientific research [7]. Therefore, health misinformation on social media urgently requires more excellent action from those working in public health research and practice. Analyzing Figures 2, 3, which illustrate the COVID-19 case trends in the United States and Canada from February 2020 to May 2021, it becomes evident that there is a notable surge in COVID-19 cases during December 2020 and January 2021. This was also the time when the Pfizer-BioNTech COVID-19 Vaccine and Moderna COVID-19 vaccine were discovered and eventually approved by the FDA. The primary reason both countries experienced a spike in COVID-19 cases during this two-month window (December-January) could be because of the Christmas Holidays/Winter Break and the New Year [6]. During this period, more individuals come into social contact with each other through public events and social gathering interactions with family and friends. One possible theory posits that the upswing in COVID-19 cases during this timeframe can be attributed to a significant segment of the population not adhering to social distancing measures. In contrast, Canada saw another substantial increase in COVID-19 cases during March, April, and May 2021, while the United States did not witness a corresponding shift in case numbers during that period. The primary reason why Canada experienced its third wave of COVID-19 is because of the development of the novel variant B.1.1.7, which first originated in the United Kingdom [11]. It is shown that 5154 of the new cases in March were transmissible variants [11]. It is also possible that Canada experienced this spike in COVID-19 due to the seasonal change from the winter to the spring/summer months. This seasonal change drives more individuals to go outside and attend more social gatherings and thus break the governed rules and social distancing restrictions. Figures 4, 5 examine the number of people who received the COVID-19 vaccination dose between January 2021 and May 2021. It can be seen that in the first few initial months, there were many doubts and hesitancy regarding vaccines in the earlier stages of vaccine circulation, but over time, the confidence in the vaccine and the availability may have increased, increasing the number of doses [12]. In the U.S., after April, however, the number of doses began to decline due to the possibility of public health misinformation. According to Figure 5, 91% of the accounts on Twitter are unverified, and only 9% of the accounts are verified. 91% accounts for around 187,533 accounts on Twitter. However, the data do not relate to how COVID-19 misinformation is spread, but it is known that a large portion of the unverified accounts are controlled by bots. According to a study cited for reference, approximately 9%-15% of active Twitter accounts are believed to be automated bots. In 2019, the same study identified roughly six billion fraudulent accounts [13]. On social media platforms, automated accounts operated by bots are designed to disseminate specific information through various tactics [14]. These tactics encompass endeavors such as attempting to make certain topics trend and artificially inflating the reach of discussions, which may involve the creation of numerous accounts with overlapping purposes. Additionally, they engage in activities such as generating, soliciting, or purchasing fake interactions, executing large-scale or aggressive tweeting, and following other users strategically. Bots also employ hashtags in a manner considered spammy [13]. Consequently, these bots can significantly contribute to the proliferation of false information concerning the COVID-19 vaccine. Studies have identified numerous bot accounts on Twitter, particularly within communities where sources with low credibility and suspicious videos are prevalent [13]. Thus, there is evidence that bots play a role in spreading misinformation on social media.

Table 1 depicts COVID-19-related most relevant hashtags out of 200 that were analyzed. Due to so many myths, as discussed earlier in this paper, many occurrences of anti-vaccine hashtags were expected. It was found that there was a significant amount of searches about COVID-19 vaccine topics, but there were limited occurrences of anti-vaccine and vaccine misinformation linked hashtags on Twitter. After investigating more, it was found that many social media platforms lock the use of such hashtags and terms associated with spreading misinformation. Looking at the table, it is still evident that around 113,855 total tweets were there for anti-vaccine-related hashtags. This number is not huge, but it also shows many people are interacting with such tweets, and there is still some misinformation spread through such posts in the media.

**Figure 8:**
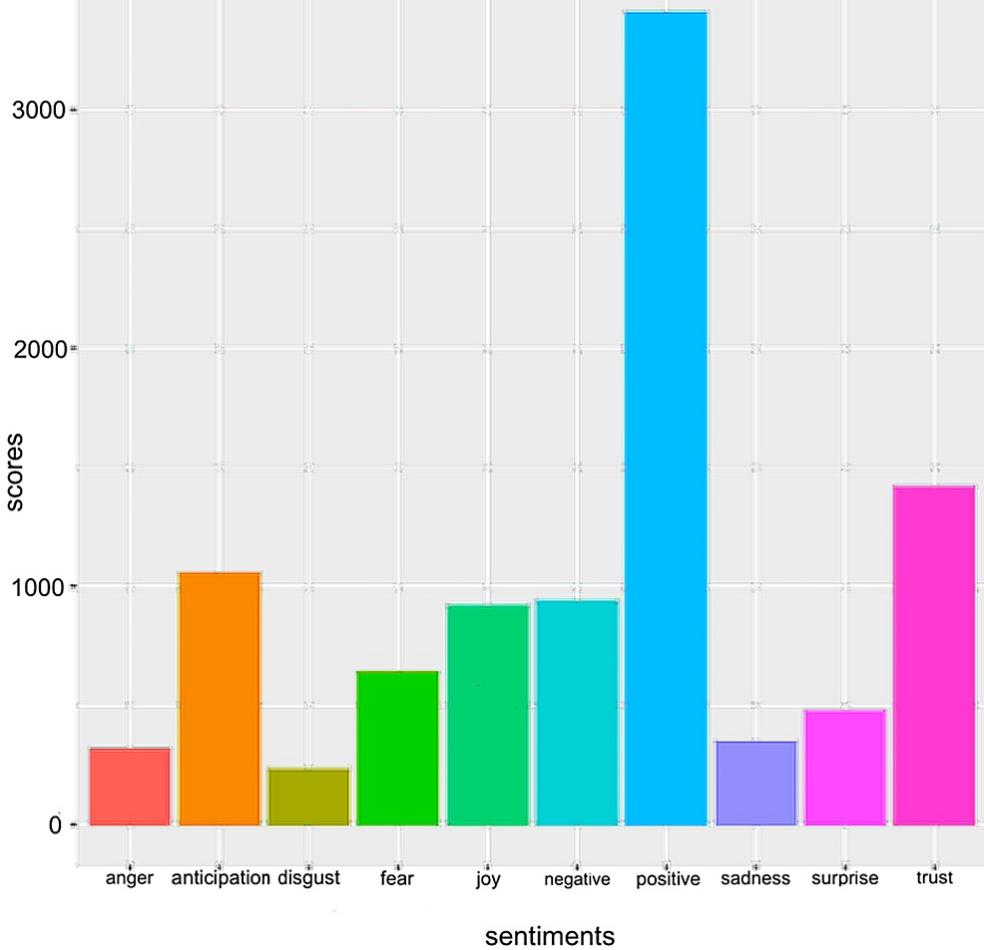
Sentiment scores from individuals about the COVID-19 vaccine on Twitter.

A sentiment scores analysis on how people feel about the COVID-19 vaccine on Twitter has been depicted as seen in Figure 8. When the COVID-19 vaccine came out in December, a wide range of emotions among the general population was observed through Twitter. A lot of people did have a positive reaction towards the COVID-19 vaccine. However, many felt emotions such as anger, disgust, fear, adverse reactions, and sadness. There was a low amount of trust in the COVID-19 vaccine as well. Thus, this shows that there has been a spread of misinformation regarding the COVID-19 vaccine when the vaccine was first developed, as many did not trust it and had an adverse reaction.

Figure 6 shows a timeline of standard search terms related to anti-vaccine ideology on Twitter. Google trend analysis showed a total of 100 standard search terms being used worldwide that are related to COVID-19 anti-vaccine ideology, out of which only five were the most popular terms, as reported in Figures 6, 7. The others were excluded from the list due to the extremely low rate of occurrence. Figure 7 shows the rate of occurrences of only two search terms, “covid-vaccine side effects” (red) and “covid-vaccine deaths” (blue), in different periods from February 2020 to May 2021.

One common trend is seen in that the frequency of these terms increased (a spike in the graph) around December 2020, which is also when the COVID-19 vaccine was approved. Thus, it is evident that the spread of misinformation has led to delays in the population not being able to accept the vaccine and trust the government as the previous graphs show that many people did not get COVID-19 vaccine doses around the early stages of the COVID-19 vaccine came out. Moreover, as the graph shows, a large group of individuals were also against the administration of the COVID-19 vaccine as they were actively seeking information about “anti-vaccine” ideology.

In Figure 7, it is seen that most people have searched on Google “Is covid vaccine safe?”. This high volume of searches indicates that many individuals in the population lack trust in the efficacy of the COVID-19 vaccine and are highly fearful despite the information given out by individuals and parties with a high level of credentials, such as the government. “Anti-vaccine” is also another highly searched topic on the internet as people are looking for valid reasons not to get vaccinated.

As of May 26, 2021, a total of 168,866,145 people have been infected with COVID-19, resulting in 3,506,304 deaths and 150,476,302 recoveries [15]. In the presence of a future global pandemic, a variety of methods can be used to reduce the spread of public health misinformation, which causes a lapse in judgment within the general public on what is the correct health measure that needs to be taken. A few methods include improving health literacy, using the internet and Google search engine as a collaborative tool with physicians, strengthening the signal of source quality found online, and promoting public figures to spread increasingly accurate information on sustainable health measures [15,16].

There are some limitations to the study, including that it remains unclear to the extent to which our explanations are concrete. Enough data needs to be collected to prove the correlation between the spread of misinformation of anti-vaccine ideology and the rise of COVID-19 cases. For some of my data, enough information was not available as to which location the false tweets were spreading from to prove that a specific location has a rise in COVID-19 cases due to the false tweets from that certain location.

Secondly, we could not confirm which accounts from all the unverified accounts (91%) were bots. Sometimes, bots can be programmed to increase the spread of misinformation. Enough resources were unavailable to implement an AI to detect the number of bot accounts on Twitter. Thus, the data and the results might be skewed as the spread of misinformation could be higher than seen in the results.

Thirdly, access to online resources and databases was limited to making a firm conclusive statement. To ascertain the extent of COVID-19 cases potentially impacted by anti-vaccine sentiments, several key data points need consideration. These include the frequency of anti-vaccine hashtag use in specific regions with lower vaccination rates and higher infection numbers, the presence of Twitter bot accounts, and the precise percentage of COVID-19 cases influenced by anti-vaccine beliefs. To better understand the correlation between anti-vaccine ideologies and COVID-19 cases, it is essential to collect or conduct surveys and gather responses from the public. These surveys should focus on reasons for vaccine hesitancy or refusal, thereby enhancing the depth and accuracy of research findings.

While this paper acknowledges certain limitations that preclude it from presenting definitive evidence about the correlation between misinformation spread on social media and the surge in COVID-19 cases, its findings remain invaluable for subsequent social media analyses. There's little doubt that social media profoundly influences public opinion. Consequently, the dissemination of false information on such platforms can pose significant public safety risks. Therefore, there's an imperative need to conduct more research exploring the primary sources of misinformation in social media platforms, the group of people receiving this information and their attitude and subsequent response to it, the number of anti-vaccine hashtags used versus the change in COVID-19 cases in a specific period and more. Social media entities should bolster their efforts in pinpointing and countering misinformation, potentially employing machine learning tools or collaborating with fact-checkers. The insights gleaned from this research undoubtedly stand to enrich related studies in the future.

## Conclusions

Coronavirus has made a significant impact on people’s lives worldwide. Ever since January 2020, all the governments around the globe, as well as pharmaceutical companies, have tried to find a vaccine to help tackle the exponential causes of COVID-19 and death rates. There has been extensive research put into determining the effectiveness of these vaccines. However, vaccines have been controversial on the internet and, more precisely, on various social media platforms. On social media, there have been growing amounts of anti-vaxxers, people who do not have trust in the vaccine, fear of dangerous side effects, and groups who believe in conspiracy theories and myths. After extensive research on various social media platforms, Twitter was the platform where a vast amount of misinformation regarding the COVID-19 vaccine was spreading, leading to a rise in COVID-19 cases around January-March 2021. This is evident through my findings discussed above. Moreover, it was found that on Twitter, there is a higher rate of occurrence of hashtags related to anti-vaccine; however, any sort of anti-vaccine hashtags is blocked by the social media platform, which prevents the spread of misinformation to an extent. The critical thing is that social media did not block such hashtags in the initial stages of spreading misinformation, which must have contributed to the rise in COVID-19 cases. Once the hashtags were blocked, the spread of misinformation lowered, and more people started to get the vaccine. Sentiment analysis of about 207,006 tweets demonstrated that while the majority of users had a positive reaction toward the COVID-19 vaccine; however, there were a significant number of people who were skeptical and had a negative response toward getting the vaccine. Finally, analyzing Google trends proved that the anti-vaccine ideology was getting very popular from December 2020; this contributed to an increase in the spread of misinformation and led to fewer people getting vaccines when the COVID-19 vaccines were first approved by the FDA around December. This also highlights the fact that there is a presence of public mistrust toward the vaccine. If such a scenario arises in the future, lessons should be learned now so that public health misinformation spread can be reduced next time. Some ways to do that would be to block the use of hashtags and posts by social media personnel, improve literacy, use Google to work collaboratively with healthcare professionals to put accurate information online, strengthening the source of quality of the information presented online, and having public figures on social media promoting accurate information on health and safety measures.
